# When the Usual Becomes Unusual: A Closer Look at Mycobacterium marinum Infections

**DOI:** 10.7759/cureus.62210

**Published:** 2024-06-12

**Authors:** Ioannis Kyriazidis, Myrto Trakatelli, Georgia-Alexandra Spyropoulou

**Affiliations:** 1 Department of Plastic and Reconstructive Surgery, Papageorgiou General Hospital, Thessaloniki, GRC; 2 Second Department of Dermatology and Venereology, School of Medicine, Faculty of Health Sciences, Aristotle University of Thessaloniki, Thessaloniki, GRC; 3 Department of Plastic Surgery, School of Medicine, Faculty of Health Sciences, Aristotle University of Thessaloniki, Thessaloniki, GRC

**Keywords:** hand infection, clinical presentation, therapy, diagnosis, trauma, hand, mycobacterium marinum

## Abstract

*Mycobacterium marinum* (*M. marinum*) is a slow-growing bacterium predominantly found in aquatic environments. While not highly virulent, it can cause skin and soft tissue infections, often misdiagnosed due to their indolent progression.

This paper presents the case of a 42-year-old male data analyst with a chronic, ulcerated lesion on his right middle finger resulting from a minor fish tank injury. Despite multiple interventions, the lesion resisted healing for 10 months. A detailed history raised the suspicion of atypical mycobacterial infection. Despite non-diagnostic initial evaluations, combined antimicrobial therapy with minocycline and rifampicin led to complete lesion healing.

Diagnosing *M. marinum* infection remains a challenge due to its nonspecific presentation. Key diagnostic criteria include resistance to standard antibiotics, history of exposure to aquatic environments, and potential contamination. While cultures are positive in 70-80% of cases, false negatives can occur, necessitating reliance on patient history and histology. Treatment involves combination antibiotics, with the prognosis generally favorable when treated early.

This case underscores the importance of considering *M. marinum* in the differential diagnoses of chronic skin lesions and the significance of targeted therapy.

## Introduction

*Mycobacterium marinum* (hereafter *M. marinum*) is a slow-growing, acid-fast, environmental mycobacterium commonly found in fresh and saltwater environments. Its biology is complex and has been extensively studied. *M. marinum* is a facultative intracellular bacterium that can infect macrophages and other phagocytic cells, leading to a persistent infection[1].

Despite its low virulence, it can cause infections in humans, particularly in those with impaired skin barriers[2]. These typically present as skin and soft tissue infections, such as nodules, papules, and ulcers, and are often misdiagnosed as other skin conditions, such as cellulitis or pyoderma, due to their slow growth and indolent clinical course. Moreover, instances of chronic granulomatous tenosynovitis post-*M. marinum* infection have been documented[3]. The diagnosis is often delayed, leading to prolonged treatment and poor outcomes[1].

The primary mode of transmission is through direct skin contact with contaminated water, manifesting as cutaneous and/or subcutaneous lesions, which may be solitary or multiple, nodular, or ulcerated, predominantly observed on the extremities of the upper limbs, including fingers and hands[1]. Individuals who are at an increased risk of infection include those with a history of exposure to fresh and saltwater environments, like aquarium hobbyists and fish handlers, as well as those with compromised skin barriers, such as those with eczema or other skin conditions[4].

In addition to direct skin contact, *M. marinum* can also spread through lymphatic drainage, leading to the clinical appearance of linear sporotrichoid lesions[5], that can be particularly challenging to treat, as they often require prolonged courses of antimicrobial therapy and can result in significant morbidity[1].

We present a compelling case that exemplifies the challenges and considerations associated with *M. marinum* infections, shedding light on its diagnostic and therapeutic intricacies.

## Case presentation

**Figure 1:**
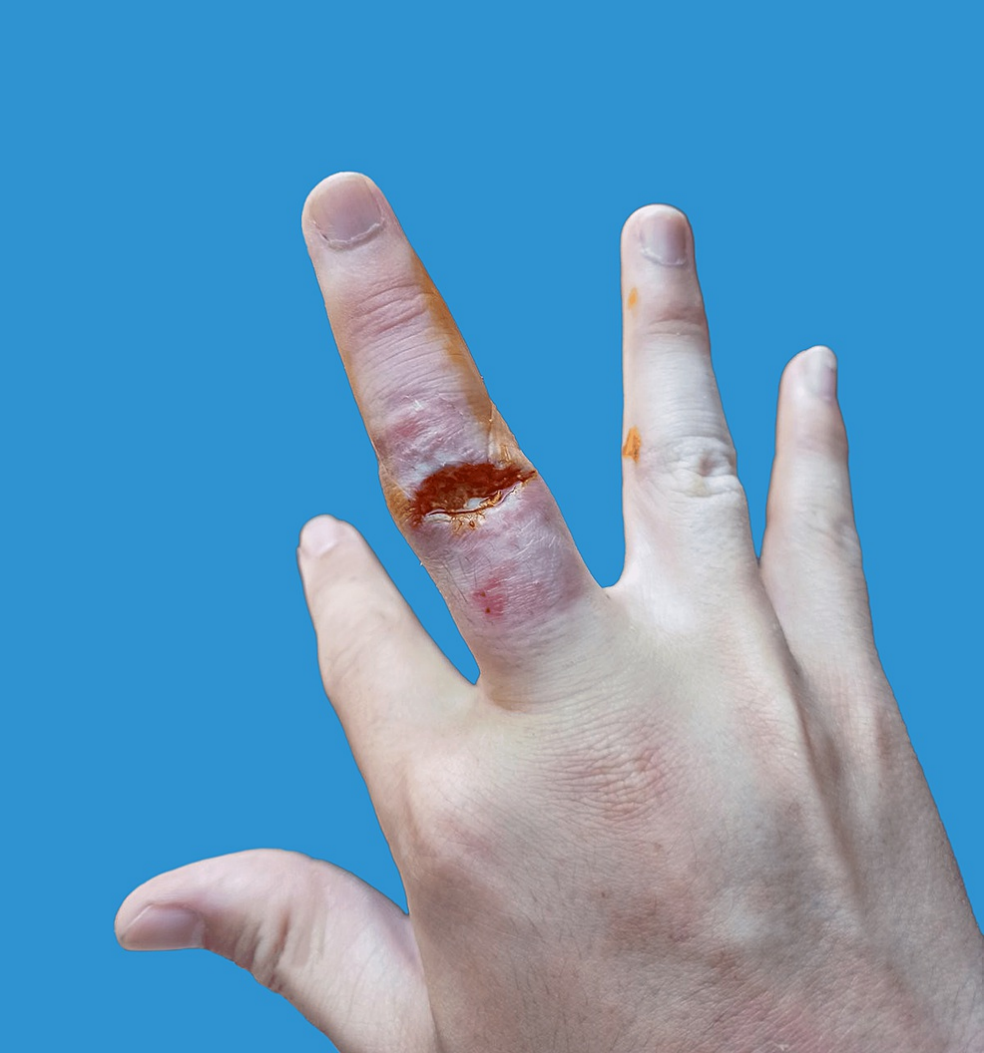
Ulcerative lesion over the PIPJ manifesting self-discharge of pus, captured approximately two weeks post-injury by the patient. PIPJ: proximal interphalangeal joint

A 42-year-old male, right-hand-dominant data analyst, with no significant past medical history presented with a chronic, ulcerated lesion located on the dorsum of the proximal phalanx of his right middle finger. This lesion emerged around 10 months prior after the patient sustained a minor cut while cleaning a fish tank. Despite the lesion's self-dehiscence shortly after the trauma (Figure 1), it had persistently resisted healing. He had attempted multiple therapeutic interventions including topical steroid ointments, courses of oral flucloxacillin, regenerative ointments, cryotherapy, multiple wound debridements, and even a failed direct closure, all to no avail.

**Figure 2:**
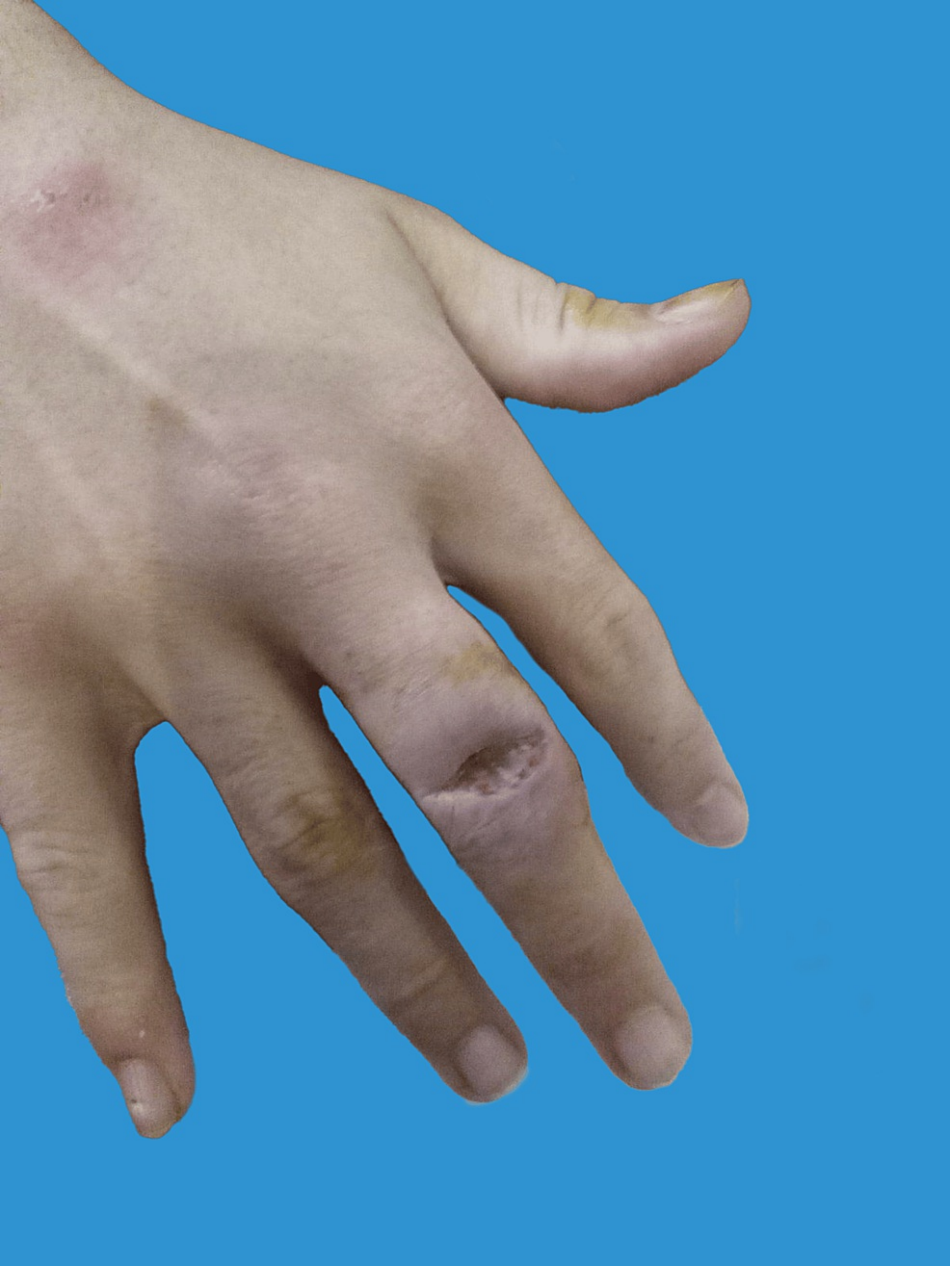
Clinical presentation of the lesion during initial assessment in the outpatients' clinic. It is characterized by a well-defined ulcerative defect, approximately 1 cm in diameter. The central area displays necrotic tissue with purulent exudate, lacking surrounding erythema or warmth, indicative of the persistent nature of M. marinum infection.

Upon clinical examination, the lesion was characterized as a well-defined ulcerative defect, approximately 1 cm in diameter, surrounded by an erythematous, elevated margin (Figure 2). 

**Figure 3:**
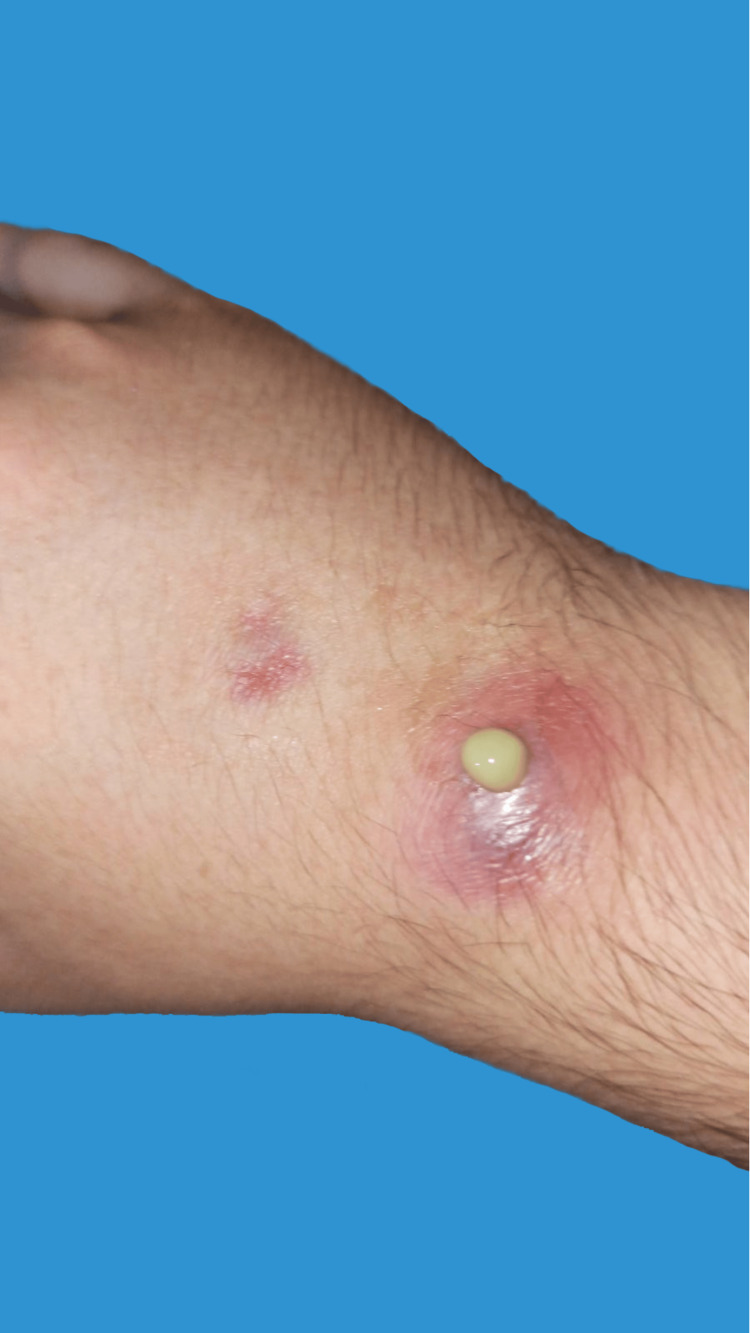
During the initial presentation, the patient was displaying two erythematous nodules expressing pus, identified as sporotrichoid lesions, on the dorsum of the proximal hand and wrist.

The center of the lesion was necrotic with purulent exudate, but notably lacked surrounding erythema or warmth. Additionally, there were two tender erythematous nodules on the dorsum of the proximal hand and wrist that were self-discharging pus (Figure 3). 

Motor and sensory functions of the hand and digits were intact, and no axillary lymphadenopathy was detected. Furthermore, the patient denied systemic symptoms such as fever, night sweats, or weight loss.

**Figure 4:**
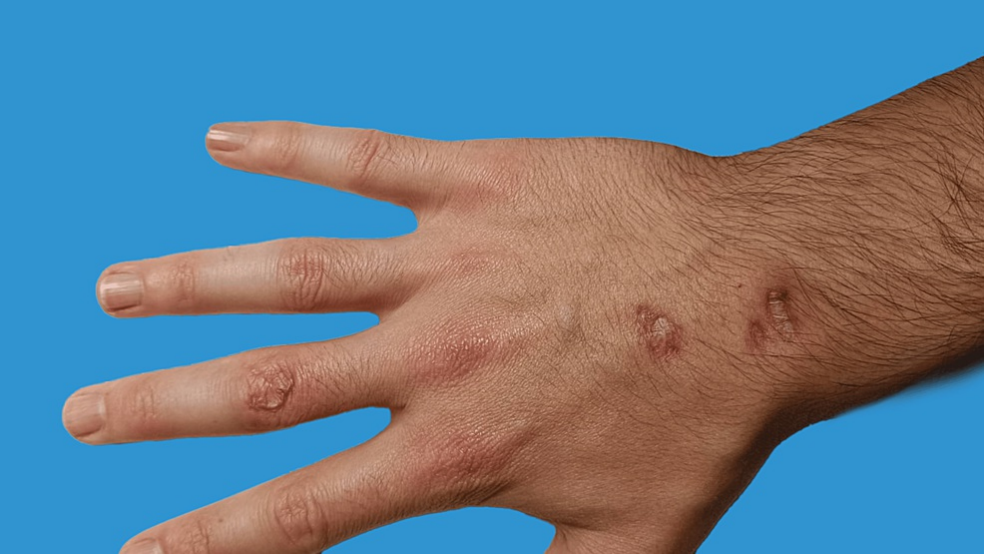
Image taken several weeks post-initiation of a prolonged course of antibiotic therapy with minocycline and rifampicin, exhibiting substantial recession of the infection and significant healing.

Radiological evaluation via MRI did not show osteomyelitis neither joint involvement but revealed soft tissue swelling. Despite the non-diagnostic outcomes from the subsequent soft tissue biopsy and wound cultures, a heightened level of suspicion for an atypical mycobacterial infection was engendered, primarily influenced by the patient's detailed hobbyist history. This led to the initiation of a three-month combined antimicrobial therapy with minocycline and rifampicin. Over this therapeutic period, the patient experienced a consistent reduction in pain and swelling. The lesion exhibited marked improvement and eventually healed completely (Figure 4) with no recorded recurrence during an eight-month follow-up period, with the patient presenting a full active range of movement to the digit and hand.

## Discussion

Cutaneous infection due to *M. marinum* is relatively infrequent, and its diagnostic journey often proves elusive due to its subtle and nonspecific manifestation. Typically, there's a pronounced gap between symptom onset and etiologic agent identification[5]. The incubation period typically spans fewer than four weeks; however, it can extend significantly, with some case reports documenting periods of up to nine months[6]. 

A heightened diagnostic suspicion is typically anchored on a triad: a cutaneous lesion showing minimal response to standard antibiotics, a history of exposure to aquatic environments (common among aquarium enthusiasts and fish farmers)[5], and a background of potential contamination.

Previous findings indicate that cultures are positive in approximately 70-80% of cases[1,3]; thus, negative cultures in the diagnosis of *M. marinum* infections, though not commonplace, represent a significant challenge in clinical practice. The slow growth rate of *M. marinum* combined with its unique metabolic needs can contribute to suboptimal growth in standard culture media, potentially leading to false-negative results [6], as in this case. Furthermore, prior antibiotic treatments, even if ineffective for *M. marinum*, can further reduce the bacterial load, making culture detection even more challenging. In cases where cultures remain negative despite high clinical suspicion, next-generation sequencing (NGS) is an emerging diagnostic tool that can aid in identifying slow-growing atypical mycobacteria like *M. marinum* from clinical specimens[7]. NGS allows for rapid, culture-independent detection of mycobacterial DNA sequences that may be present in low abundance, providing an alternative method when routine cultures fail to yield a definitive diagnosis [1]. However, the absence of a positive culture result can create diagnostic ambiguity, which underscores the importance of maintaining a high clinical suspicion. In many scenarios, the lesions caused by *M. marinum* don't follow a distinct pattern.

Diagnostic clarity may be further clouded by a broad differential diagnosis that includes other non-tuberculous mycobacteria, sporotrichosis, and several non-infectious conditions[5]. Treatment modalities are influenced by guidelines and generally advocate for a combination antibiotic regimen lasting until lesion healing, followed by 1-2 additional months[1,8]. Notably, *M. marinum* is sensitive to a range of medications but resistant to others[8]. 

Thus, a synergistic approach involving histological assessment and mycobacterial studies (comprising both cultures and molecular diagnostics if available) is pivotal for its accurate identification.

Treatment regimens, like those opted for in our presented case, are typically tailored to individual patient needs, considering both lesion severity and potential underlying conditions. Surgical interventions play a pivotal role in certain cases, particularly extensive lesions[1]. The prognosis for *M. marinum* cutaneous infections remains optimistic, especially when treated before any deeper tissue or bone involvement [1,8]. This is clearly evidenced by the prompt treatment and favorable outcome of our reported case.

## Conclusions

 In light of this case and the supporting literature, it's evident that *M. marinum* infections, though not highly virulent, can pose significant diagnostic and therapeutic challenges. A thorough patient history, especially concerning exposure to aquatic environments, combined with a high degree of clinical suspicion, can guide clinicians towards a more accurate and timely diagnosis. This case serves as a reminder of the importance of considering atypical pathogens in the differential diagnosis of chronic skin lesions and the value of persistent and targeted antimicrobial therapy for achieving favorable outcomes.
